# Decorin levels and cardiometabolic function after endurance exercise

**DOI:** 10.3389/fphys.2025.1546370

**Published:** 2025-07-08

**Authors:** Antônio Alves de Fontes-Junior, Cesar Augustus Zocoler de Sousa, Leandro Barbosa de Oliveira, Ana Paula Renno Sierra, Maria Fernanda Cury-Boaventura

**Affiliations:** ^1^ Interdisciplinary Post-Graduate Program in Health Sciences, Cruzeiro do Sul University, São Paulo, Brazil; ^2^ School of Physical Education and Sport, University of São Paulo, São Paulo, Brazil

**Keywords:** decorin, exercise, metabolism, myokine, performance

## Abstract

**Introduction:**

Decorin is secreted from myotubes in response to exercise and plays a vital role in repairing and regenerate skeletal and cardiac muscle. The role of decorin levels in cardiometabolic function after endurance exercise was investigated.

**Methods:**

Fifty-six male amateur runners, aged 30 to 55 years, participated in this study. Plasma decorin levels were determined and cardiopulmonary exercise test (CPET) was performed using a breath-by-breath system before and after the marathon.

**Results:**

Correlations between decorin levels and CPET parameters were assessed using Spearman’s rank correlation test. Runners were categorized into percentiles based on decorin concentrations, and group differences were analyzed using the Kruskal-Wallis test followed by Dunn’s post hoc multiple comparisons.We observed impairments in peak VO_2_ and in the Oxygen Uptake Efficiency Slope (OUES) following the race. Plasma decorin levels increased approximately two-fold immediately after the race. Post-race decorin concentrations were positively correlated with maximum speed (km/h), VO_2_ (mL/kg/min), VE/VCO_2_, VE/VCO_2_ slope, OUES, and the rate of change in VE/VCO_2_ and VE/VCO_2_ slope. Runners with plasma decorin levels below the 25th percentile exhibited significantly lower OUES, while those above the 75th percentile demonstrated higher maximum speed, VE/VCO_2_, rate of change in VE/VCO_2_ slope, and VCO_2_ compared to individuals in the lowest quartile.

**Discussion:**

Overall, decorin levels were associated with several CPET parameters, suggesting that runners with varying decorin concentrations may exhibit distinct respiratory and/or metabolic profiles. The potential influence of an elevated VE/VCO_2_ slope on cardiometabolic responses in runners with higher decorin levels warrants further investigation.

## 1 Introduction

Endurance exercise can lead to transient impairments in cardiac function, characterized by left (LV) and right ventricular (RV) dysfunction. These alterations may result from reductions in blood volume, decreased β-adrenergic receptor responsiveness, and/or myocardial ischemia or damage (Martinez et al., 2021). Moreover, endurance exercise induces skeletal muscle damage, triggering the release of various myokines that play key roles in tissue repair, angiogenesis, fat oxidation, and glucose uptake—factors that collectively influence cardiometabolic function (Jin et al., 2024; Ashcroft et al., 2024).

Decorin, a member of the small leucine-rich proteoglycan (SLRP) family, is an extracellular matrix (ECM) protein secreted by myotubes in response to exercise. Research on the ECM highlights its essential role in muscle development, growth, and regeneration. The ECM is composed of collagen, elastin, proteoglycans, and glycoproteins and serves as a reservoir for growth factors such as transforming growth factor-beta (TGF-β) and myostatin, which are known to interact with ECM-related proteoglycans (Iozzo, 1999; Kresse and Schönherr, 2001). Decorin binds to multiple types of collagen, thereby regulating fibril formation and stabilization within the ECM (Miura et al., 2006; Brandan and Gutierrez, 2013) It also plays a pivotal role in cell growth through its interactions with cell surface receptors—including receptor tyrosine kinases and toll-like receptors—and by modulating the activity of key growth factors such as IGF-1, TGF-β1, and TGF-β2 (Vu et al., 2018).

In skeletal muscle, decorin has been shown to modulate cell proliferation, differentiation, and autophagy, supporting muscle regeneration. It also antagonizes myostatin, a negative regulator of muscle growth, by sequestering it within the ECM and inhibiting its activity via activation of the SMAD2/3 signaling pathway—ultimately reducing protein degradation in skeletal muscle (Kishioka et al., 2008; Willoughby et al., 2022; El Shafey et al., 2016). While increased decorin levels have been reported following endurance exercise, and its role in skeletal muscle function is increasingly recognized, its involvement in cardiometabolic modulation in this context remains unclear.

Emerging evidence suggests that decorin may also influence angiogenesis through interactions with various growth factors, cytokines, and cell surface receptors, with effects ranging from promotion to inhibition of endothelial cell survival (Järveläinen et al., 2015). *In vitro* study has observed a direct cardiocytoprotective effect of decorin, as well as the evaluation of two polymorphisms of decorin demonstrated the involvement of this gene on cardiovascular function (Kunnas et al., 2016; Gáspár et al., 2020). Furthermore, decorin appears to play a role in lipid metabolism in both adipose and skeletal tissues. In adipose tissue, it may function as a receptor for resistin and influence adipocyte metabolism and proliferation (Daquinag et al., 2011).

We hypothesized that decorin, secreted from myotubes in response to exercise, may contribute to the prevention of cardiometabolic dysfunction following endurance activity. This hypothesis is based on evidence from the literature suggesting that decorin interacts with cell surface receptors and growth factors in the extracellular matrix (ECM), thereby promoting skeletal and cardiac muscle regeneration, enhancing angiogenesis, and increasing fat oxidation in muscle tissue. In this study, we investigated the role of decorin response in cardiometabolic function after endurance exercise.

## 2 Materials and methods

### 2.1 Subjects

Seventy-four amateur Brazilian male marathon finishers (aged 30–55 years) were recruited in accordance with Sierra et al. (2022). Exclusion criteria included the use of medications for cardiac, metabolic, pulmonary, or renal conditions; alcohol or drug use; and the presence of systemic arterial hypertension or any liver, kidney, metabolic, inflammatory, or neoplastic disease. Inclusion criteria required prior participation in at least one marathon or half marathon and a minimum weekly training distance of 30 km.

All participants were informed of the study procedures and potential risks, and provided written informed consent. The study protocol was approved by the Ethics Committee of Cruzeiro do Sul University (approval number: 29046919.4.0000.8084), in accordance with the Declaration of Helsinki.

From an initial pool of 74 amateur runners, 12 were excluded for not undergoing cardiopulmonary exercise testing (CPET) before or after the marathon, and 11 were excluded for missing decorin measurements at one or both time points. The final sample comprised 51 runners.

**FIGURE 1:**
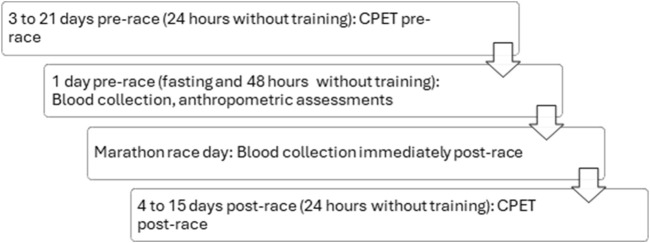
Experimental design of the study.

Cardiopulmonary exercise testing (CPET) was conducted at two time points: baseline (3–21 days prior to the marathon) and post-race (4–15 days following the event), with all assessments performed after a minimum of 24 h without physical training. Venous blood samples were collected at baseline (under fasting conditions) and immediately upon completion of the marathon. Anthropometric measurements were obtained 1 day before the race, following a fasting period of at least 4 h (Figure 1).

Anthropometric assessments, including total body mass (kg), height (cm), and body mass index (BMI, kg/m^2^), were performed 1 day prior to the marathon at the University, following the guidelines of the International Society for the Advancement of Kinanthropometry (ISAK). Results are reported as mean ± SEM. Body composition (fat mass and fat-free mass percentages) was evaluated using bioelectrical impedance analysis (Biodynamics Corporation, United States, model 310e). Details of the marathon race protocol have been previously described by Sierra et al. (2022).

### 2.2 Cardiopulmonary exercise testing (CPET)

The CPET was performed using a treadmill protocol (TEB Apex 200, TEB, São Paulo, Brazil) with adjustable speeds ranging from 0 to 24 km/h and inclines from 0% to 35%. The test was conducted with a fixed incline of 1%, starting at 8 km/h and increasing by 1 km/h every minute until the participant reached maximal exhaustion. Expired gas analysis was conducted using a breath-by-breath system (Quark CPET, Cosmed, Rome, Italy). Cardiac activity was continuously monitored throughout the test using a standard 12-lead electrocardiogram to evaluate any exercise-induced cardiac changes. The CPET parameters collected included: total exercise time, maximal speed, oxygen consumption (VO_2_, mL/kg/min), carbon dioxide production (VCO_2_, L/min), respiratory quotient (RQ), ventilation (VE, L/min), VE/VO_2_, VE/VCO_2_, partial pressure of end-tidal oxygen (PETO_2_, mmHg), partial pressure of end-tidal carbon dioxide (PETCO_2_, mmHg), percentage of fat and carbohydrate oxidation, and the oxygen uptake efficiency slope (OUES). These parameters were assessed at the anaerobic threshold (AT), respiratory compensation point (RC), and peak exercise.

### 2.3 Measurement of plasma decorin

Blood samples (10 mL) were collected in vacuum tubes containing an anticoagulant (0.004% EDTA). Samples were centrifuged immediately to isolate plasma, which was stored at −80°C for subsequent decorin level analysis. Immediately after the race blood samples were collected from a research area located close to the finish line and sent to Cruzeiro do Sul University for plasma collection and storage. Plasma decorin concentration was measured by enzyme-linked immunosorbent assay (DuoSet-ELISA R&D systems, United States) as described by De Sousa et al. (2021). The intra-assay precision for plasma decorin concentration was <10% as described by the manufacturer’s protocol.

### 2.4 Statistical analyses

Statistical analyses were performed using GraphPad Prism version 9. Data distribution normality was assessed using the Kolmogorov-Smirnov test, and non-parametric methods were employed due to rejection of normality. Descriptive statistics for general and training characteristics were presented as mean ± standard error of the mean. Comparison of cardiometabolic parameters and decorin levels before and after the marathon utilized the paired non-parametric Wilcoxon test. Correlations between decorin levels and CPET parameters were assessed using the Spearman correlation test. The samples were distributed in percentiles based on decorin concentration (<25th, 25th-50th, 50th-75th, >75th), and differences between groups were evaluated using the Kruskal–Wallis test followed by Dunn’s post-test for multiple comparisons. The rate of change in CPET parameters was calculated as the ratio of post-race to pre-race values, to assess the relative increase or decrease. Effect size for the Kruskal–Wallis test was determined using eta-squared (η^2^), calculated as η^2^ = (H − k + 1)/(n − k), where *H* is the Kruskal–Wallis statistic, *k* is the number of groups, and *n* is the total sample size. Statistical significance was considered at a level of p < 0.05 for all analyses.

## 3 Results

The baseline and training characteristics of the participants were as follows: age, 41.1 ± 7 years; weight, 75 ± 10 kg; height, 1.72 ± 0.06 m; BMI, 24.7 ± 4.3 kg/m^2^; fat mass percentage, 22% ± 5%; fat-free mass, 58 ± 6 kg; marathon race time, 255 ± 43 min; 10 km race time, 47 ± 6 min; and weekly training volume, 55 ± 15 km.

Cardiopulmonary exercise testing (CPET) parameters are presented in Table 1, highlighting performance impairments observed at the anaerobic threshold (AT), respiratory compensation point (RC), and peak. We demonstrated an impairment in the time test, VO_2_, RQ, PETO_2_, VE/VO_2_, fat and carbohydrate oxidation (% and g/min) at AT; VO_2_, VCO_2_, RQ, VE, PETCO_2_ and VE/VO_2_ at RC and VO_2_ at peak and in the Oxygen Uptake Efficiency Slope (OUES) (Table 1). Marathon race time was negatively correlated with both maximum speed and VO_2_peak (r = −0.40, *p* < 0.008 and r = −0.36, *p* < 0.018, respectively) pre- and post-race.

**TABLE 1:** Cardiopulmonary exercise testing parameters.

CPTE parameters	At		RC		Peak	
	Before	After	Before	After	Before	After
Time (min)	3.1 ± 1.0	2.8 ± 0.5*	7.1 ± 1.9	7.3 ± 2.1	5.0 ± 1.3	4.8 ± 0.8
Speed (km/h)	11 ± 1.9	11 ± 1.4	19 ± 3.0	18 ± 3.0	20 ± 3.1	20 ± 3.3
VO_2_ (mL/kg/min)	32.7 ± 5.4	31.6 ± 3.5**	51.3 ± 8.9	48.1 ± 6.9***	54 ± 8.9	50 ± 6.8***
VCO_2_ (L/min)	1899 ± 297	1940 ± 352	3702 ± 510	1940 ± 352*	4085 ± 601	4028 ± 518
RQ	0.79 ± 0.07	0.83 ± 0.09***	1.0 ± 0.05	1.0 ± 0.08**	1.1 ± 0.07	1.1 ± 0.09
VE (L/min)	57 ± 11	58 ± 13	124 ± 23	119 ± 20	118 ± 32	127 ± 20
VE/VO2	23 ± 3.1	24 ± 3.6**	32 ± 3.8	33 ± 4.1*	35 ± 5.4	35 ± 5.7
VE/VCO2	29 ± 2.5	29 ± 3.0	31 ± 3.8	31 ± 3.9	32 ± 5.0	32 ± 5.7
PETO2	90 ± 4.8	90 ± 12.6*	101 ± 12.6	102 ± 3.7*	103 ± 4.9	104 ± 3.8
PETCO2	38 ± 2.7	39 ± 3.7	36 ± 3.6	36 ± 4.1*	36 ± 4.5	36 ± 4.3
Fat ox (%)	70 ± 24	57 ± 28***	5 ± 11	2 ± 5	1.4 ± 4	0.4 ± 2
Fat ox (g/L)	0.92 ± 0.4	0.72 ± 0.35***	0.11 ± 0.3	0.04 ± 0.11	0.02 ± 0.07	0.01 ± 0.03
CHO ox (%)	29 ± 23	42 ± 27**	95 ± 12	98 ± 5	99 ± 4	98 ± 9
CHO (g/L)	0.79 ± 0.6	1.18 ± 0.78***	4.5 ± 0.8	4.4 ± 0.6	4.2 ± 1.0	4.4 ± 0.6
VE/VCO_2_ slope					28 ± 3.1	28 ± 3.3
OUES					3680	3444*

AT, anaerobic threshold; RC, respiratory compensation, VO2, oxygen consumption and VCO2, carbon dioxide production, RQ, respiratory quotient; VE, ventilation, PETO2 (mmHg), PETCO2 (mmHg), Fat ox, fat oxidation, CHO, ox, carbohydrate oxidation and OUES, Oxygen Uptake Efficiency Slope (OUES). The data are presents as mean ± DPM, of 51 runners. *p < 0.05, **p < 0.001 and ***p < 0.0001.

Plasma decorin levels increased approximately two-fold immediately after the marathon (from 109 ± 14 pg/mL to 220 ± 23 pg/mL; *p* < 0.0001). No significant correlations were observed between post-race decorin levels and baseline or training-related characteristics (data not shown).

Post-race decorin concentrations were positively correlated with maximum speed (km/h), VO_2_ (mL/kg/min), VE/VCO_2_, VE/VCO_2_ slope, and OUES (r = 0.34, *p* = 0.010), and negatively correlated with carbohydrate oxidation (%), respiratory quotient (RQ), and PETCO_2_ (Table 2). Additionally, decorin levels were positively associated with the rate of change (post-race/pre-race) in VE/VCO_2_ and VE/VCO_2_ slope, and negatively associated with the rate of change in RQ and PETCO_2_ (Table 2).

**TABLE 2:** Correlation between maximum CPET parameters post-race and absolute value and rate of change in decorin levels post-race.

CPET	Absolute value		Rate of change	
	*r*	p	*r*	p
Speed (km/h)	0.54	<0.0001	−0.029	0.84
VO_2_ (mL/kg/min)	0.31	0.033	−0.22	0.12
VE/VCO2	0.55	<0.0001	0.44	0.0014
VE/VCO2 slope	0.36	0.009	0.40	0.004
CHO oxidation (%)	−0.40	0.0025	−0.19	0.19
RQ	−0.63	<0.0001	−0.39	0.0041
PETCO2	−0.59	<0.0001	−0.33	0.017

CPET, cardiopulmonary exercise testing; RQ, relative quotient; CHO, oxidation, carbohydrate oxidation.

**FIGURE 2:**
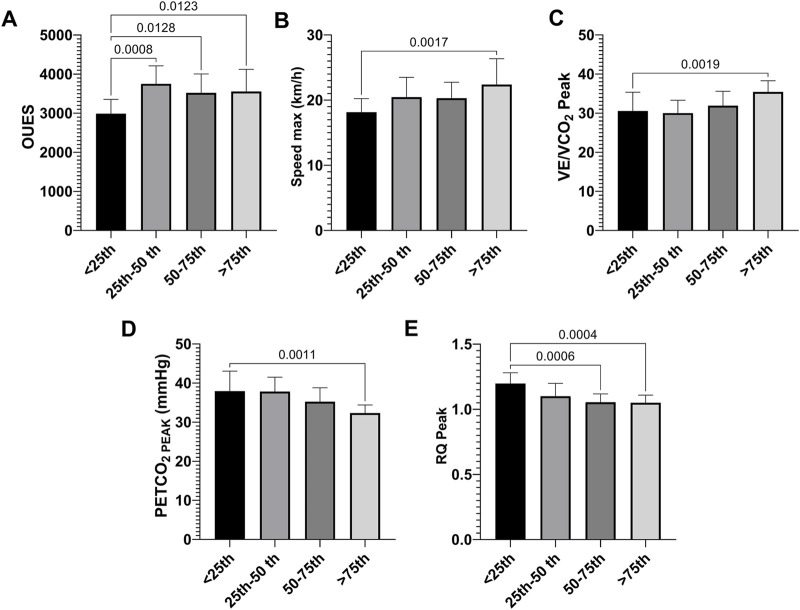
Cardiopulmonary exercise testing parameters in runners with varying decorin levels after the race. Data are presented as mean ± SD for OUES **(A)**, maximum speed **(B)**, VE/VCO_2_ **(C)**, and PETCO_2_ **(D)** and RQ **(E)** at peak exercise. Runners were grouped by decorin levels into four categories: below the 25th percentile (n = 13), between the 25th and 50th percentiles (n = 11), between the 50th and 75th percentiles (n = 14), and above the 75th percentile (n = 13).

Participants were stratified into percentiles based on post-race decorin concentrations (<25th, 25th–50th, 50th–75th, and >75th percentiles). Runners with decorin levels below the 25th percentile exhibited significantly lower OUES (η^2^ = 0.26; Figure 2A). Conversely, those with decorin levels above the 75th percentile demonstrated higher maximum speed (η^2^ = 0.19) and VE/VCO_2_ (η^2^ = 0.19) compared to individuals in the lowest quartile (Figures 2B,C). Furthermore, runners in the highest decorin percentile showed significantly lower PETCO_2_ (η^2^ = 0.30) and RQ (η^2^ = 0.34) than those in the lowest percentile (Figures 2D,E), suggesting a distinct respiratory and metabolic phenotype associated with elevated decorin levels.

**FIGURE 3:**
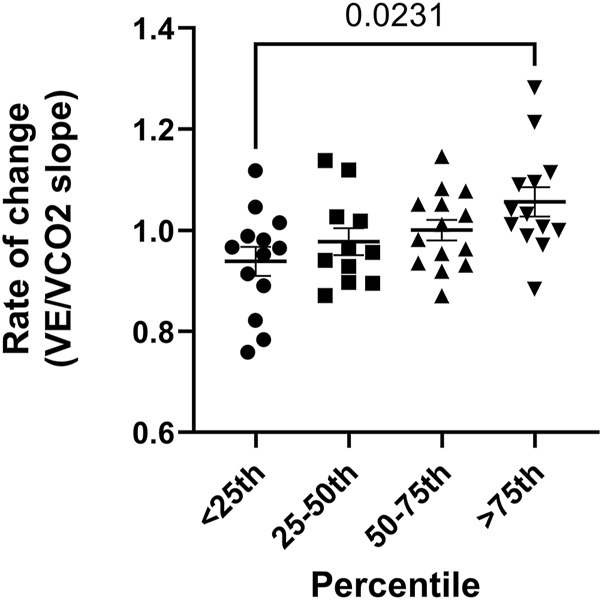
Rate of change in VE/VCO_2_ slope in runners with varying decorin levels after the race. Data are presented as individual values and mean ± standard error of the mean (SEM) for changes in OUES (ratio of post-race to pre-race values) in runners grouped by decorin levels: below the 25th percentile (n = 13), between the 25th and 50th percentiles (n = 11), between the 50th and 75th percentiles (n = 14), and above the 75th percentile (n = 13).

Finally, the rate of change in VE/VCO_2_ slope and VCO_2_ (post-race/pre-race) varied by decorin concentration. Runners with the lowest post-race decorin levels showed an increase in the VE/VCO_2_ slope (η^2^ = 0.10) and VCO_2_ (η^2^ = 0.22) compared to those with the highest levels (Figure 3). No significant differences were observed in the rate of change for speed, VE, VO_2_, PETCO_2_, or OUES across decorin percentiles.

## 4 Discussion

Marathon racing induces impairments on cardiometabolic function post-race, as evaluated by CPET, primarily demonstrated by the reductions in VO_2peak_ and OUES. After the race, plasma decorin concentration was positively associated with maximum speed (km/h), VO_2_ (mL/kg/min), VE/VCO_2_, VE/VCO_2_ slope, and OUES. Runners with plasma decorin levels below the 25th percentile exhibited significantly lower OUES and runners with elevated levels of decorin exhibited higher maximum speed and rate of change in the VE/VCO_2_ slope suggesting improved metabolic function.

The cardiometabolic system involves several organs including the heart, lungs, skeletal muscle, endothelium and adipose tissues. Skeletal muscle contraction triggers the release of various skeletal muscle-derived humoral factors such as myokines, metabolites, non-coding regulatory RNAs and exosomes to modulate metabolic and cellular functions for cardiometabolic adaptation or tissue repair. The release of exercise-induced peptides is dependent on the type of exercise and training protocol, resulting in a variety of myokines stimulated by endurance exercise (Piccirillo, 2019; Domin et al., 2021).

Previous study from our group observed an increase in decorin levels after the marathon running (de Sousa et al., 2021). Skeletal muscles contain chondroitin/dermatan sulphate proteoglycans, such as decorin, in the ECM. Decorin modulates myogenesis, protein synthesis, angiogenesis and lipid metabolism, and has anti-inflammatory and anti-fibrotic properties by interacting with cell surface receptors and modulating growth factors, thus contributing to cardiometabolic tissue repair or adaptations (Vu et al., 2018).

In skeletal muscle, decorin directly interacts with SMAD-2/3 complex, inhibiting myostatin and thereby reducing the degradation of proteins in skeletal muscle. Myostatin acts on the activin receptors (type I and II), promoting the phosphorylation and activation of SMAD proteins. SMAD-2 and SMAD-3 form a complex with SMAD-4, inducing the transcription of catabolic genes (Kishioka et al., 2008; Willoughby et al., 2022; El Shafey et al., 2016). Additionally, a decrease in decorin levels reduces sensitivity to TGF-β dependent inhibition of myogenesis (Iozzo, 1999; Kresse and Schönherr, 2001).

The mechanical and metabolic stress induced by repetitive contractions of muscle fibers, including energy ADP/ATP ratio challenges, loss of calcium homeostasis, oxidative stress, increased calpain activity, and inflammation also contribute to muscle injury (Hody et al., 2019). Skeletal muscle damage is accompanied by mitochondrial dysfunction as well as ischemia/reperfusion (I/R) injury in the myocardium, leading to a transient cardiometabolic dysfunction. I/R injury is a result of restriction and subsequent restoration of blood supply to an organ promoting the accumulation of reactive oxygen species that contribute to cell death and tissue damage.

Previous reviews have suggested that decorin possesses antioxidative properties that help attenuate oxidative stress and ischemia/reperfusion (I/R) injury (Szabó et al., 2020). Researchers have also explored the role of small leucine-rich proteoglycans—such as glycan and decorin—in protecting myocardial cells from I/R injury, proposing a cardioprotective effect mediated by Toll-like receptor 4 (TLR-4) signaling. This mechanism involves the activation of survival kinases and increased nitric oxide (NO) production (Gáspár et al., 2020; Gáspár et al., 2016). Studies investigating two polymorphisms of the *decorin* gene have demonstrated its involvement in cardiovascular risk (Kunnas et al., 2016; Gáspár et al., 2020). Animal studies further indicate that decorin contributes to ventricular remodeling following acute myocardial infarction and is essential for appropriate fibrotic development in infarcted myocardium (Takemoto et al., 2002; Zimmerman et al., 2001; Weis et al., 2005). Additionally, gene therapy using *decorin* has shown potential in attenuating cardiac dysfunction, positioning it as a promising therapeutic target for myocardial infarction (Li et al., 2009).

However, the interaction of decorin with other proteins in various physiological and pathological contexts may either increase or decrease cardiovascular risk (Vu et al., 2018). Despite its known roles, studies on decorin in exercise-induced cardiometabolic adaptations remain limited. In the present study, we observed correlations between decorin levels and several cardiopulmonary exercise parameters, including maximum speed (km/h), VO_2_ (mL/kg/min), VE/VCO_2_, VE/VCO_2_ slope, and OUES. However, decorin concentration was specifically associated with the rate of change (post-race/pre-race) only for VE/VCO_2_ and VE/VCO_2_ slope. Evidence from *in vitro* and animal studies also supports the role of decorin as a key extracellular signaling molecule in the regulation of cardiac autophagy, metabolism, and function, particularly under nutrient-deprivation conditions (Gubbiotti et al., 2018). TGF-β downregulates metabolic adaptation to exercise by reducing insulin signaling, mitochondrial activation, and enzymes involved in optimal metabolic adaptations. Decorin can interact with TGF-β, thereby avoiding metabolic dysfunction (Böhm et al., 2016). Indeed, we found that runners with the highest decorin levels exhibited a greater oxygen uptake efficiency slope (OUES), higher maximum speed, and an elevated VE/VCO_2_ slope after the race, suggesting enhanced cardiometabolic function. However, no significant differences were observed in the rate of change in speed, VO_2_ peak, or OUES, indicating that higher decorin levels did not prevent the cardiopulmonary impairments typically induced by endurance exercise.

Finally, we observed a relative increase in both VE/VCO_2_ slope and VCO_2_ in runners with the highest decorin levels post-race. A previous study reported a negative correlation between VO_2_peak and VE/VCO_2_ slope (Chaumont et al., 2023). The VE/VCO_2_ slope reflects ventilatory efficiency and may indicate an abnormal ventilatory response to exercise or increased physiological dead space (Chaumont et al., 2023). The elevated VE/VCO_2_ slope observed in runners with higher decorin levels may reflect a metabolic adaptation, potentially characterized by more efficient oxygen utilization, greater running speed, or a ventilation-perfusion mismatch. This imbalance could contribute to decreased PETCO_2_ and RQ, accompanied by an increased VE/VCO_2_ slope. However, further studies are needed to clarify the relationship between decorin levels and changes in PETCO_2_, RQ, and VE/VCO_2_ slope.

Overall, decorin levels were associated with several cardiopulmonary exercise testing (CPET) parameters, including maximum speed, OUES, VO_2_, and the VE/VCO_2_ slope, as well as post-race changes in the VE/VCO_2_ slope. These findings suggest that runners with low and high decorin levels may exhibit distinct respiratory and/or metabolic profiles However, decorin levels do not appear to prevent cardiometabolic dysfunction induced by endurance exercise. The potential impact of an increased VE/VCO_2_ slope on cardiometabolic responses in runners with elevated decorin levels warrants further investigation.

### 4.1 Limitation of the study

One limitation of this study is the exclusive inclusion of male amateur runners, which restricts the generalizability of the findings to females. The use of peripheral plasma decorin as a proxy for local muscular or cardiac tissue response is also a limitation, as it does not directly reflect tissue-specific activity. Finally, CPET was performed between 3–21 days pre-race and 4–15 days post-race which may minimize the cardiopulmonary dysfunction after race, in spite we observed this impairment.

## Data Availability

The raw data supporting the conclusions of this article will be made available by the authors, without undue reservation.
